# A Nursing Mess and Its Teaching

**Published:** 1916-03-11

**Authors:** 


### THE MATRONS' AND SISTERS' DEPARTMENT.

### A Nursing Mess and its Teaching.

We are indebted to the assistant matron for the following notes on the messing arrangements for the nursing staff at the 1/5th Northern General Hospital, Leicester:—

The 1/5th Northern General Hospital, Leicester, was mobilised on August 5, 1914. For the first few months the mess and other arrangements for the nursing staff presented great difficulties. All the cooking for the above was carried out in the hospital kitchen, which was staffed by the R.A.M.C. Their work was very heavy, as the kitchen accommodation was quite inadequate to deal with the cooking for 600 patients, officers, R.A.M.C. Company, and nursing staff; it was therefore impossible for any mess sister to efficiently control waste or to have full justice done to the cooking for her own department, as twenty-one meals per day were cooked and served from the one kitchen. In May 1915 the extension of the hospital to 1,140 beds necessitated the building of nurses' quarters, cubicles, and kitchen, two dining-rooms being subsequently added to supersede a nurses' dining-room which would no longer comfortably seat the increased staff. At this time I was fortunate enough to be appointed assistant matron, thus becoming responsible for the mess arrangements. We have now a nursing staff of 182, with twenty-four maids. The kitchen staff consists of one cook, an assistant cook, with three maids; each of the two dining halls has three permanent waitresses, and the cubicle maids assist in waiting at table.

### Time-table for Meals.

Breakfast, 6.30 a.m., both dining halls; breakfast, 7.30 a.m., sisters in dining hall No. 1; night nurses' dinner, 8 a.m., in dining hall No. 2; lunch, 9 a.m., both dining halls; dinner, 12.30 to 1 p.m., dining hall No. 1; dinner, 1 to 1.30 p.m., dining hall No. 2; teas from 4.30 to 5.30 p.m., both dining halls; supper, 8 p.m., both dining halls.

### The Daily Menu.

*Breakfast.*—Tea, bread, butter, marmalade, bacon and tomatoes, with liver sometimes. Porridge every other morning, owing to difficulties in the milk supply. *Lunch.*—Coffee, bread, butter, and dripping. *Dinner.*—Either beef or mutton, pork, stew, beef-steak pies, or fish. Potatoes, and, four times a week, a second vegetable. Sweets, either stewed fruits, banana cream, trifle, milk puddings, jam tarts, or boiled suet puddings. *Tea.*—Tea, bread, butter, jam, alternating with scones and cake. *Supper.*—Soups on alternate days, with either sausages, cottage pies, rissoles, fish cakes, tripe, boiled vegetables, or curry; puddings as for dinner.

### The Mess Money allowed to each member of the nursing staff is 15s. a week, of which 12s. 6d. is paid to the matron for food, while 2s. 6d. is retained for washing. When any member of the staff is away on leave she retains her mess money. The money is passed on to me for the payment of the accounts. After the monthly accounts are paid a balance sheet is prepared and shown to all the staff. For the period from January 1 to December 31, 1915, we were able to refund a considerable sum to members of the nursing staff who were here during that time; but to enable this to be done I have been careful to dispose of the swill and dripping to the best advantage. Owing to the increase in food prices, I fear it will not be possible again to return as much as for the last year.

### Its Aim, Arrangements, and Results.

The details given regarding the menus show that it has been possible to provide, within the limits allowed by the amount charged, considerable variety, although, of course, there can be few luxuries; the aim has been to supply good plain food, well cooked, in ample quantities. The arrangement by which sisters, nurses, and V.A.D. members take their meals in common has helped considerably to make it possible to provide satisfactory service at a reasonable expense.

